# Editorial: Neuroanatomical considerations and advances in approaches for cerebral arteriovenous malformations

**DOI:** 10.3389/fneur.2026.1743430

**Published:** 2026-03-09

**Authors:** Feres Chaddad-Neto, Raphael Wuo-Silva, Juan Carlos Ahumada-Vizcaíno, Glaucia Suzanna Jong-A-Liem, Luis Angel Canache Jiménez

**Affiliations:** 1 Department of Neurology and Neurosurgery, Universidade Federal de São Paulo, São Paulo, SP, Brazil; 2 Department of Neurosurgery, Hospital Beneficência Portuguesa de São Paulo, São Paulo, SP, Brazil

**Keywords:** cerebral arteriovenous malformations, endovascular procedures, microsurgical interventions, neuroanatomical advances, vascular anomalies

Cerebral arteriovenous malformations (AVMs) continue to represent one of the most fascinating and challenging cerebrovascular entities (1–6). Defined by direct connections between arteries and veins without an intervening capillary bed, these lesions embody the intersection between neuroanatomical complexity and therapeutic innovation. This Research Topic, “*Neuroanatomical considerations and advances in approaches for cerebral arteriovenous malformations*,” was conceived to integrate anatomical knowledge with emerging surgical and endovascular strategies, aiming to advance both scientific understanding and clinical management of these vascular anomalies. This Research Topic includes three original contributions, each addressing a distinct facet of AVM biology, diagnosis, and treatment.

The first original paper by Wang et al. provide a compelling molecular perspective on the pathogenesis of AVMs. The authors investigated the regulatory role of Insulin-Like Growth Factor 2 mRNA Binding Protein 2 (IGF2BP2) in vascular development, aiming to elucidate epigenetic mechanisms underlying AVM formation. Through an integrative bioinformatic approach combining RNA sequencing (RNA-seq) and methylated RNA immunoprecipitation sequencing (MeRIP-seq), they identified IGF2BP2 as a significantly downregulated gene in AVM tissues compared with normal cerebral vessels. Functional experiments using endothelial cell cultures and zebrafish models demonstrated that IGF2BP2 deficiency leads to abnormal angiogenesis and disrupted cerebrovascular development. Mechanistically, the study revealed that IGF2BP2 regulates LGALS8 expression by modulating mRNA stability through N6-methyladenosine (m6A) modification. Loss of LGALS8 further impaired angiogenesis *in vitro* and induced cerebrovascular malformations *in vivo*. This work highlights the importance of epigenetic regulation in cerebrovascular biology, positioning IGF2BP2 as a key modulator of vascular integrity. By uncovering the IGF2BP2–LGALS8 axis, the study opens new avenues for understanding the molecular basis of AVMs and suggests novel therapeutic targets aimed at restoring normal vascular development and stability.

The second original paper by Miron et al. provides an important contribution to understanding the prognostic factors in brain AVM care by examining two distinct endpoints: early post-operative functional outcome in surgically treated patients and overall long-term survival in the full cohort. In this retrospective single-center study analyzed 191 patients treated for brain AVMs between 2012 and 2022, 79 underwent microsurgical resection; 51 presented with ruptured AVMs and complete resection was achieved in 68 cases (86.1%). Deep venous drainage was significantly associated with incomplete resection. Regarding early post-operative functional status, an unfavorable outcome was defined as mRS at discharge > 2, and female sex, admission mRS > 2, and eloquent brain location emerged as independent predictors of this endpoint. Separately, the authors assessed early neurological deterioration, defined as a worsening of mRS from admission to discharge, and found that multiple drainage veins were associated with a higher risk of such early worsening. Beyond surgical outcomes, Miron et al. evaluated overall mortality (all-cause) over long-term follow-up across the entire cohort, showing that eloquent involvement, conservative management, older age, admission mRS > 2, and comorbidities were liked to reduced survival, whereas interventional treatment (surgery, embolization, radiosurgery, or multimodal therapy) was associated with significantely better survival compared with conservative management after adjustment for age and admission neurological status. Collectively, these findings reinforce the value of clearly defining endpoints in AVM research and support an individualized, proactive therapeutic strategy that accounts for baseline neurological status and lesion complexity when balancing early functional risk against potential long-term survival benefit.

The third contribution by Chen et al. presents an innovative endovascular approach for the treatment of complex non-cavernous intracranial dural arteriovenous fistulas (DAVFs). Although distinct structurally, DAVFs and AVMs share hemodynamic and anatomical principles relevant to this Research Topic. The authors describe a novel grouting technique, which combines transvenous embolization using detachable coils and Onyx to achieve more complete and durable occlusion of these lesions. The study included 20 patients with aggressive or symptomatic intracranial DAVFs, predominantly high-grade sinus-type fistulas with cortical venous reflux (mainly Cognard IIb, IIa+b, III, and IV). The procedure involved placing two microcatheters (one for coil delivery and the other for Onyx injection) within the affected sinus or draining veins. Coils were first deployed from distal to proximal segments to create a stable scaffold, followed by the controlled injection of Onyx to fill the coil interstices and penetrate the fistulous channels, ensuring complete closure. The results were favorable, with successful embolization was achieved in all 20 patients, with complete angiographic occlusion in 95% immediately after treatment and 100% obliteration confirmed at follow-up. No recurrence, rebleeding, or need for additional interventions was observed during a 2–5-year follow-up period. This study highlights the grouting technique as a safe, durable, and highly effective endovascular strategy for managing complex intracranial DAVFs. By integrating the mechanical stability of coils with the cohesive sealing properties of Onyx, the method represents a significant advancement in neuroendovascular surgery, reducing recurrence risk and improving long-term outcomes.

In summary, this Research Topic, entitled *Neuroanatomical considerations and advances in approaches for cerebral arteriovenous malformations*, brings together a diverse set of studies that illustrate the multifaceted progress in understanding and managing vascular lesions of the central nervous system. The works presented range from endovascular innovations, as shown by Chen et al., to molecular and epigenetic discoveries revealing new regulatory pathways in vascular development, as demonstrated by Wang et al., and clinical analyses that refine prognostic assessment and therapeutic decision-making, as explored by Miron et al. Together, these contributions underscore how the integration of anatomical, molecular, and technical perspectives continues to shape modern vascular neurosurgery. By bridging fundamental science with clinical application, this Research Topic highlights the ongoing evolution toward more precise, individualized, and less invasive approaches for the treatment of cerebral arteriovenous malformations. Ultimately, the studies included here expand scientific understanding and reaffirm the importance of interdisciplinary collaboration in achieving safer procedures and improved long-term outcomes for patients with these complex vascular disorders.
